# Effect of a novel anti-CCR4 monoclonal antibody (Mogamulizmab) on skin lesions of adult T-cell leukemia-lymphoma (ATL) and its adverse skin reactions (ASR)

**DOI:** 10.1186/1742-4690-11-S1-P24

**Published:** 2014-01-07

**Authors:** Kentaro Yonekura, Tamotsu Kanzaki, Nobuaki Nakano, Masahito Tokunaga, Ayumu Kubota, Shogo Takeuchi, Yoshifusa Takatsuka, Atae Utsunomiya

**Affiliations:** 1 Department of Dermatology, Imamura Bun-in Hospital, Kagoshima, Japan; 2 Department of Hematology, Imamura Bun-in Hospital, Kagoshima, Japan

## 

We studied the effects of Mogamulizmab on ATL and its adverse reactions in 10 patients with ATL, among them 6 patients had skin lesions. Informed consent was obtained prior to study. Four out of 6 patients with cutaneous involvement showed complete response (CR), with 4 to 8 cycles of treatments. Of interest were two cases which appeared to have worsened in the early phase of treatment because of enlargement of cutaneous nodules or tumors. It, however, was found to be actually improving determined from histopathological examinations, *i.e.*, more inflammatory cell infiltration and edema with less number of lymphoma cells in the skin. Eventually, these two patients showed CR. Furthermore, 2 each patients with 4 CR patients showed no recurrence with ASR, *i.e.*, erythema and plaque, and showed reccurence without ASR. ASR were observed in 4 out of 10 patients. All these ASR fortunately subsided later. Immunohistopathological examinations revealed the infiltration of cytotoxic T-lymphocytes in the dermis. These results suggest that 1) Mogamulizmab had excellent effects (4/6) to suppress the growth of cutaneous lesions in ATL, 2) ASR might be favorable signs of the effects (2/4), and 3) ASR (4/10) were not serious. Studies of plasma and tissue levels of Mogamulizmab and T-reg cells may reveal the action mechanism of this novel anti-CCR4 agent in detail.

